# Intraosseous angiosarcoma with secondary aneurysmal bone cysts presenting as an elusive diagnostic challenge

**DOI:** 10.1186/1477-7800-5-10

**Published:** 2008-05-20

**Authors:** Lung Fung Tse, Eugene TH Ek, John L Slavin, Stephen M Schlicht, Peter FM Choong

**Affiliations:** 1 Department of Orthopaedics, St. Vincent's Hospital, Melbourne, Australia; 2 Bone and Soft Tissue Sarcoma Service, Peter MacCallum Cancer Centre, Melbourne, Australia; 3 Department of Anatomical Pathology, St Vincent's Hospital, Melbourne, Australia; 4 Department of Radiology, St. Vincent's Hospital, Melbourne, Australia

## Abstract

Angiosarcoma of bone is an exceedingly rare primary bone malignancy that can present as an aggressive osteolytic lesion. Histological diagnosis can be extremely challenging, as the pathological features often resemble that of aneurysmal bone cysts. We report an interesting and peculiar case of an intraosseous angiosarcoma that presented as a diagnostic dilemma and discuss the relevant radiological and pathologic findings.

## Introduction

Angiosarcomas are aggressive, malignant, vascular tumors derived from mesenchymal cells. They can occur in any organ of the body, however the development of intraosseous angiosarcoma is exceedingly uncommon [1]. The clinical course of disease is often aggressive and requires timely management, as the prognosis in such patients is generally poor [2]. Histologically, the features of angiosarcomas of bone can often resemble aneurysmal bone cysts [1,2]. Therefore differentiation with clinicopathological correlation is paramount when making a diagnosis and devising subsequent treatment.

## Case presentation

Mr. RJ was a 70 year old gentleman who presented with right knee pain. The pain in his leg had been present for many months and was progressively becoming more swollen to the point that he had difficulty ambulating. This was on the background of a past history of bilateral popliteal artery aneurysms that were ligated and bypassed surgically. Examination of his knee revealed a large knee joint effusion with considerable restriction in motion due to pain.

**Figure 1:**
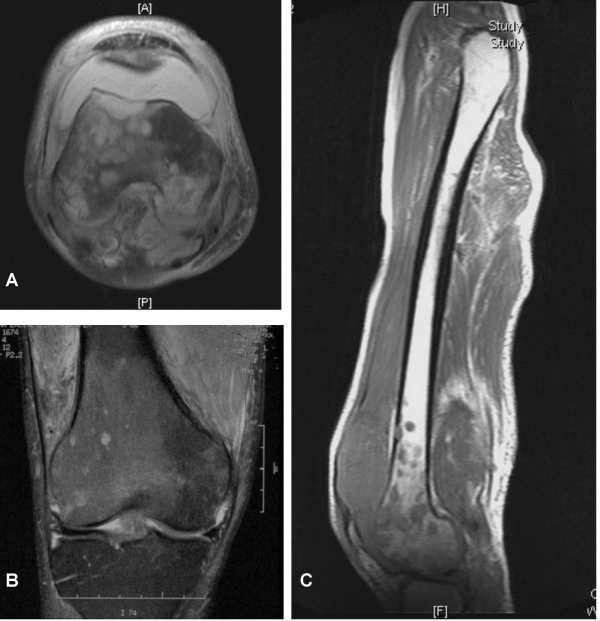
MRI demonstrating extensive medullary signal abnormality over the distal femur and proximal tibia with a large haemarthrosis. (A) T1-weighted axial, (B) T2-weighted coronal and (C) T1-weighted sagittal views of the lesion.

Radiographs of the right knee demonstrated multiple permeative lytic lesions surrounding the distal femoral condyle and metaphyseal region, resembling a "soap-bubble" appearance. Magnetic resonance imaging (MRI) of the knee revealed numerous rounded areas that were hyperintense on T2-weighted imaging in the subcortical bone of the distal femur (Figure 1). Some of the lesions exhibited a sclerotic margin and two lesions appeared to breach the cortex and extend into the soft tissues adjacent to the bone. Diffuse abnormal bone marrow signal was seen in the distal femoral diaphysis and metaphysis but not the proximal part of the femur. A large aneurysm arising from the femoral artery was evident, that extended to the popliteal artery, with MR evidence of thrombosis. Around the knee joint there was diffuse oedema and synovitis. A triple-phase bone scan of the knee was performed that showed abnormal tracer uptake in the distal right femur which was greatest in the lateral femoral condyle. The bone scan appearance was not typical for metastatic disease and there was no evidence of osteoblastic metastatic disease elsewhere. Thallium scan showed an active metabolic process occurring within the lateral femoral condyle with permeative bone lesions.

Computer tomography (CT) guided closed biopsy of the lesion was performed. The histological examination showed fibrotic changes and was deemed non-diagnostic. Because of the bizarre clinicopathological picture, an open biopsy was performed and yielded two grams of haemorrhagic soft tissue. Histological examination showed a reactive process resembling aneurysmal bone cyst formation similar to the CT percutaneous biopsy. There was no evidence in either biopsy sample to indicate malignancy or infection.

**Figure 2:**
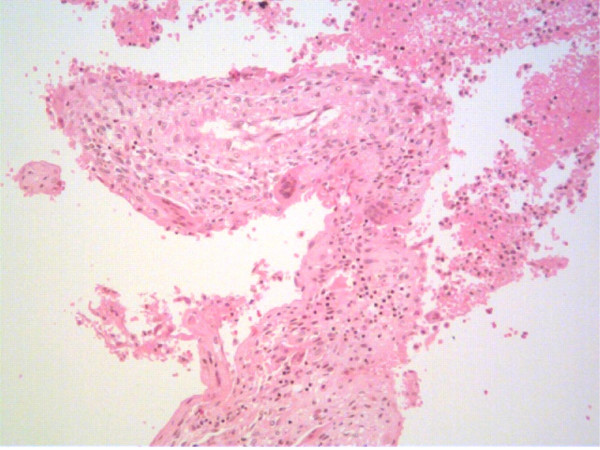
Histological section from the initial resected distal femur demonstrating cystic spaces that are lined by fibrous septa that contain areas of haemosiderin deposition and recent haemorrhage. Features were consistent with haemorrhagic aneurysmal bone cyst. No evidence of malignancy seen. (Haematoxylin and eosin staining, 10× magnification).

Following this, the patient continued to experience ongoing symptoms over the knee. Repeat MRI revealed progressive multiple, destructive bone lesions involving the right femur, with some haemorrhagic components in the soft tissue around the knee. It was decided that a wide en bloc resection of the distal femur and proximal tibia would be performed. A rotating hinge megaprosthesis (Global Modular Replacement system, Stryker, Howmedica) was used for reconstruction of a mobile knee joint. Histological analysis of the resected specimen showed cystic spaces which were lined by fibrous septa that contained blood vessels (Figure 2). Prominent, multinucleated giant cells were seen and there were areas of haemosiderin deposition and recent haemorrhage. No other accompanying neoplastic cells were seen. Therefore, given these features, a provisional diagnosis of cystic haemorrhagic aneurysmal bone cyst was made. The patient received rehabilitative exercise postoperatively, however, the knee pain was persistent.

Two months after the index operation, he presented with an acute extensive and painful haemarthrosis. Angiogram of the right leg was performed and revealed extravasating collateral branches from the profunda artery around the knee joint. Exploration and ligation of the right popliteal aneurysm and evacuation of the knee joint haematoma was performed. The patient was also anaemic, however, haematological assessment showed no underlying blood malignancies that could account for this, leaving chronic blood loss the most likely clinical cause.

**Figure 3:**
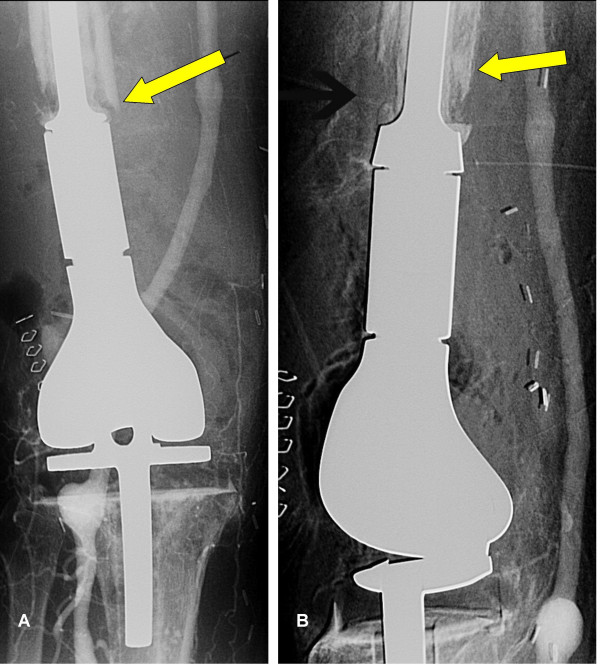
Rapid progression of the disease six months afterwards as evidence by extensive osteolysis over the bone implant junction (arrows).

**Figure 4:**
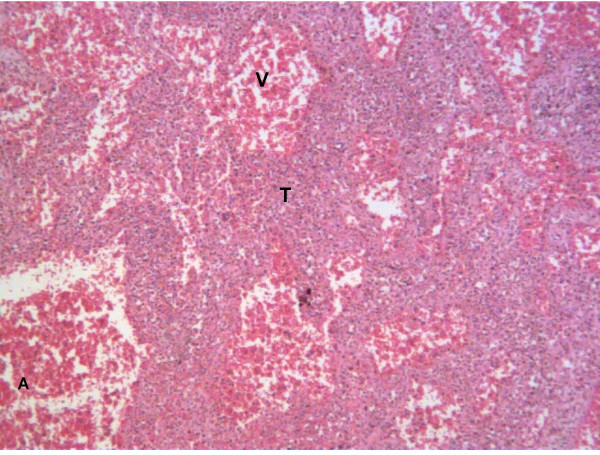
Histological section after hindquarter amputation demonstrating large dilated vascular spaces (V) lined by a dense population of atypical plump endothelial-like cells (T and arrow) which are surrounded by disorganized small diameter vascular channels. These features are consistent with angiosarcoma. (Haematoxylin & eosin staining; (A) 10× magnification, (B) 40× magnification).

The patient continued to have unremitting limb pain, and an x-ray of the knee showed progressive osteolytic destruction over the bone-implant junction (Figure 3). It was suggested that this was the result of tumour to the proximal part of the right leg. Subsequently, a hindquarter amputation was performed 5 months after the initial surgery. The macroscopic specimen was sent for expert consultation at the Mayo clinic, Rochester, MN, USA where the definitive diagnosis of angiosarcoma was made (Figure 4).

The patient's disease progressed and he subsequently developed pulmonary and intra-abdominal metastases. Lung biopsy confirmed tumor metastasis. He died of metastatic disease and sepsis one year after initial presentation.

## Discussion

Angiosarcoma is a tumour derived from mesenchymal cells, which have undergone angioblastic differentiation, resulting in the formation of blood vessels [2]. Primary angiosarcoma of bone is extremely rare and accounts for less than 1% of all angiosarcomas [1,2]. From data from the Surveillance, Epidemiology and End Results study, only 36 (1.4%) of 2,627 primary bone sarcomas were classified as angiosarcomas [1]. The clinical course of disease is aggressive, as demonstrated by this current case study. The initial closed and open biopsies were not definitive. Due to the unusual nature of this case, an open biopsy was performed that suggested the presence of a benign vascular process. It was because of ongoing symptoms and bony destruction that we elected to resect the distal femur, and from representative tissue sections, the diagnosis of aneurysm bone cyst was made. From initial imaging, both the thallium scan and MRI, suggest isolated distal femoral disease with no radiological abnormalities evident elsewhere in the skeletal system. Micro-seeding of the disease was not shown.

Reports of cardiac and pulmonary angiosarcoma highlight the difficulty in making the initial diagnosis due to the relatively non-specific clinical course and apparently normal radiological findings [4,5] High-grade lesions usually exhibit intense vascularisation and can be associated with bleeding diathesis and haematoma formation [6,7]. In our case, the large haemarthrosis and persistent knee pain was attributed to the extravasation of blood from abnormal vessels around the knee joint and neovascularisation in the proximal tibia.

MR imaging findings of angiosarcoma are relatively non-specific. Characteristically, they demonstrate low signal intensity on T1-weighted images and heterogeneously intermediate to high signal intensity on T2-weighted images [8]. In our case, the tumor showed mixed signal intensity relative to muscle on T2-weighted FSE MR images. High signal intensity on T2-weighted images may reflect necrotic tissue within the tumour, and low signal intensity can correlate with slight vascular channel formation [9] Nakazona *et *al, reported that angiosarcoma arising in soft tissue demonstrate early and continuous enhancement on Gadolinium-enhanced T1-weighted SE MR images [8]. Moreover, the tumor also may show diffusely heterogeneous enhancement on Gadolinium-enhanced T1-weighted SE MR images with fat suppression, which corresponds to areas of tumour infiltration. Although surgical resection with a wide margin of normal tissue is needed to treat angiosarcoma, it may be considered difficult to determine a definitive margin solely on the basis of MR imaging findings [9].

As seen in this case, the histologic features of aneurysmal bone cyst can mimic that of angiosarcoma. On histopathology, aneurysmal bone cysts (ABCs) are blood-filled cystic cavities that are lined by a thick, fleshy membrane that has an endothelial-like inner layer. Nearly 50% occur as a secondary lesion in another tumor. These lesions include giant cell tumor, chondroblastoma, osteoblastoma and some malignant tumors [1]. Often, the secondary lesions may be actually larger than the associated primary tumor. Total destruction of the metacarpal bone has been reported, implying a selected group of aggressive cases [10]. However, the majority of ABCs have good responses to primary treatment [11,12]. In our case, the patient suffered from persistent knee pain despite the initial apparently wide surgical resection of the tumor. Furthermore, the clinical course deteriorated progressively which is not compatible to the disease progression of ABCs. The diagnosis of angiosarcoma can be confirmed by immunohistochemical staining for endothelial cell markers such anti-CD31 [13-15]. However, morphology with clinicopathologic correlation tends to be a better guide than available special techniques [16-18]. Although ABCs may occur at any age, primary ABCs rarely occurs after 30 years of age, and it is exceptional after 50 years [12]. Thus, secondary ABCs with underlying malignancy should be ruled out especially in adult with rapid progression of disease as demonstrated in our case.

The 5-year survival rate for patients with angiosarcoma is reported to be 12% to 31% [1,2,18,19] Reports suggest that patients with lesions less than 10 cm in diameter survive longer than those with larger lesions [2,19] One case has been reported of primary angiosarcoma of the T8 vertebra, which was successfully managed with en bloc spondylectomy and postoperative chemotherapy and the patient remained disease-free at 5 years [20]. Effectiveness of adjuvant chemotherapy remains unknown. Budd *et al *reported that doxorubicin-ifosfamide chemotherapy produces a modest response rate in angiosarcoma [19]. Timely diagnosis and surgical resection may offer the patient potential cure, but in the large majority, the prognosis is dismal.

## Conclusion

Angiosarcoma of bone is a rare primary bone sarcoma. The diagnosis is often delayed because of the non-specific clinical presentation and radiological features. Clinicopathological correlation is of paramount importance to differentiate aneurysmal bone cyst from the aggressive type of vascular malignancies. Timely surgery is potentially curative although prognosis of most of the cases is often dismal.

## Competing interests

The authors declare that they have no competing interests.

## Authors' contributions

The authors equally contributed to the preparation and drafting of this manuscript.
